# Supplementary material for: The causal relationship between the human gut microbiota and pyogenic arthritis: a Mendelian randomization study
Source: Front Cell Infect Microbiol. 2024 Nov 26;14:1452480. doi: 10.3389/fcimb.2024.1452480 (PMC11629706; doi:10.3389/fcimb.2024.1452480)
Supplement: Supplementary file 1 [file DataSheet1.zip › mendelian test/ebi-a-GCST90027732.csv_forest.pdf]

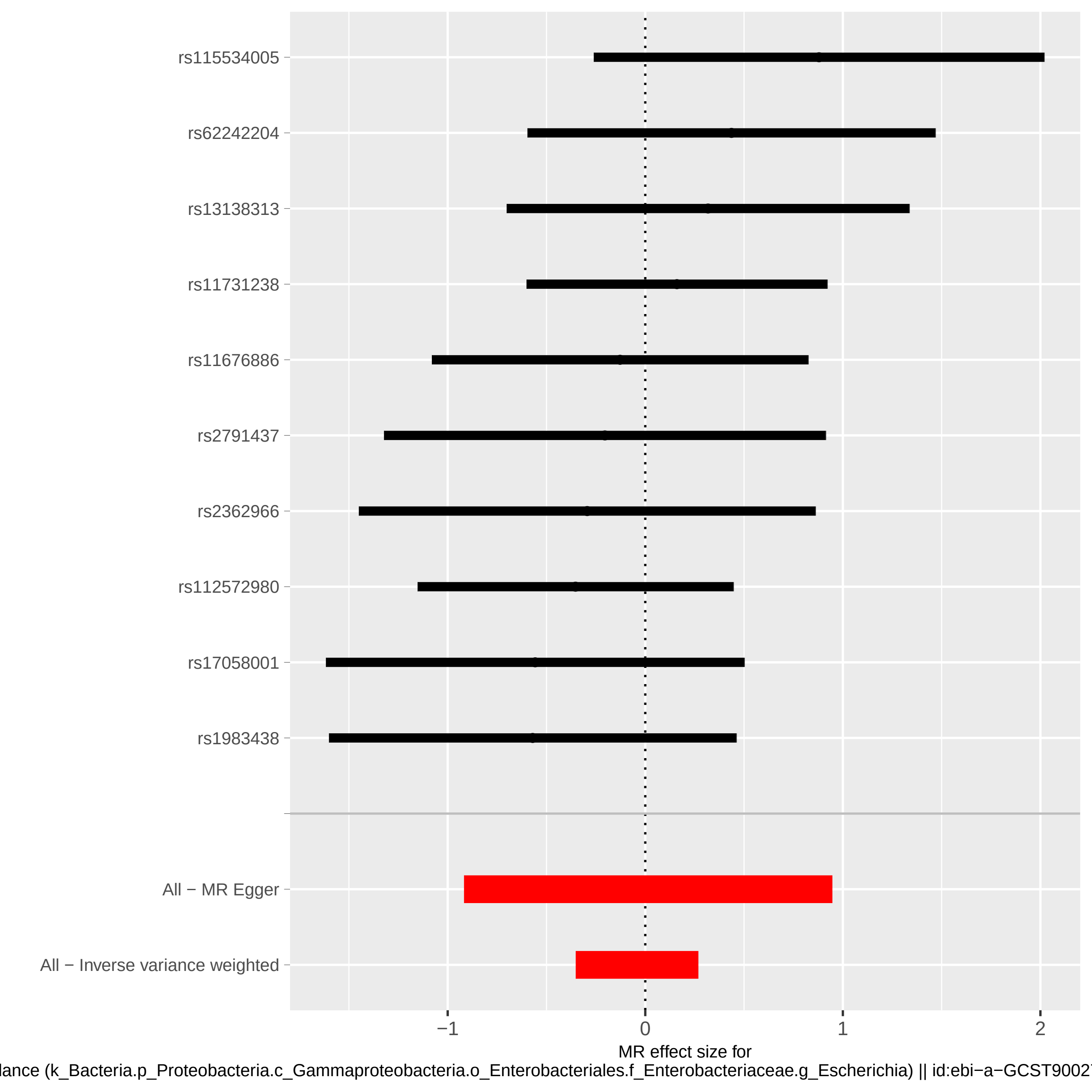
rs115534005
rs62242204
rs13138313
rs11731238
rs11676886
rs2791437
rs2362966
rs112572980
rs17058001
rs1983438
All − MR Egger
All − Inverse variance weighted
−1
0
1
2
MR effect size for
lance (k_Bacteria.p_Proteobacteria.c_Gammaproteobacteria.o_Enterobacteriales.f_Enterobacteriaceae.g_Escherichia) || id:ebi−a−GCST9002
